# Reflections from the ‘Hold the door open’ project: Inviting older adults across the UK to shape dissemination of health research findings

**DOI:** 10.1111/hex.13928

**Published:** 2023-11-29

**Authors:** Taru Silvonen, Carmel McGrath, Anne Murray, Hannah Christensen

**Affiliations:** ^1^ Health Protection Research Unit in Behavioural Science and Evaluation at the University of Bristol, Department of Population Health Sciences Bristol Medical School Bristol UK; ^2^ The National Institute for Health and Care Research Applied Research Collaboration West (NIHR ARC West) at University Hospitals Bristol and Weston NHS Foundation Trust Bristol UK; ^3^ School of Health and Social Wellbeing, Faculty of Health and Applied Sciences University of West England Bristol UK; ^4^ Independent collaborator Belfast United Kingdom of Great Britain and Northern Ireland

**Keywords:** co‐design, dissemination, engagement, older adults, patient and public involvement, vaccines

## Abstract

**Background:**

This project sought to expand patient and public involvement (PPI) practices to the development of research finding dissemination with people aged 55+ years. The project is innovative due to its UK‐wide approach and use of PPI to plan better ways to share findings of health research with older adults, extending PPI beyond research project initiation to support dissemination activities.

**Objective:**

The aim of this study is to understand how to develop effective public engagement activities with older adults to disseminate findings of health research. We hope to promote greater inclusivity and advance our understanding of this demographic.

**Methods:**

This project combined three approaches: (i) an online questionnaire to ask what activities older adults enjoy; (ii) online planning workshops seeking public contributors' input in event planning and (iii) community events to share research findings and raise awareness of PPI. Activities were carried out in Cardiff, Belfast, Glasgow and Tewkesbury.

**Results:**

The planning workshops clarified that in‐person activities and offering options for activities were important. Based on feedback from our contributors, all our events focused around a talk and question and answer session. Other short activities included light exercise and a writing activity.

**Discussion:**

Our multiphase approach helped us develop informative activities that reflected the questionnaire results and the feedback from the workshops, as we tailored our events to each location. A phased approach allowed both researchers and contributors to gradually deepen their understanding.

**Conclusion:**

Further awareness raising is needed to develop the role older adults currently hold in health research activities. Working closely with existing communities can help broaden diversity.

**Patient or Public Contribution:**

Thirty‐three public contributors helped facilitate this project. Two of these also contributed to this article by writing a reflection of their experiences, one of whom also provided feedback for the article.

## 1 INTRODUCTION

Despite the aging population in the United Kingdom,^1^ people aged 55+ years have been underrepresented in health research,^2^ emphasising the importance to focus on this demographic group. Patient and public involvement (PPI) can support further inclusion of older adults in health research by increasing awareness of ways to get involved beyond conventional research participant roles.^3^, ^4^ PPI is a mandatory element of research funded by the National Health Service,^5^ the National Institute for Health and Care Research and the Health Research Authority.^6^ Effective inclusion of patients, service users, carers and members of the public in the broad spectrum of health research is important for ethical reasons, as well as to support the generation of research ideas and accuracy of research.^7^, ^8^ Limited direct feedback from people aged 55+ makes it challenging to create appropriately tailored ways to involve older adults in health research‐related activities.

There are opportunities for PPI at every stage of the research process.^9^ Current recommendations emphasise the importance of embedding PPI throughout research projects.^10^, ^11^, ^12^ Yet, PPI activities usually involve people at the early stages of projects seeking feedback on topic guides or wording on questionnaires.^2^, ^13^, ^14^ While there are some examples of PPI in dissemination,^15^ further developing the ways researchers share their findings beyond conventional academic outputs is needed.^16^ Involving the public in the planning of dissemination activities through collaborations that draw on people's ‘experiential knowledge and expertise’^17^ also supports public engagement—sharing research with the general public. Public involvement differs from public engagement; however, the two can also complement each other.^18^

This paper is a reflective account of a public involvement project aiming to involve and co‐design health research dissemination activities with older adults (aged 55+) from diverse backgrounds. The objectives of the PPI project are: (i) exploring what kind of dissemination activities older adults might enjoy; (ii) designing engagement activities together with older adults and (iii) holding engagement events that would reach older adults from diverse backgrounds to disseminate our research findings. This PPI project ‘Hold the door open’—involving older adults from diverse backgrounds in health research—is innovative due to its focus on older adults as a broader age group and using PPI activities to shape dissemination. This paper aims to reflect on the approach used to involve older adults in co‐developing dissemination activities and to engage older adults through community events. This is done by considering what challenges we met during the project and what lessons we learnt in the process.

## 2 METHODS

### 2.1 Study approach

This PPI project was funded by the National Institute for Health Research (NIHR) Health Protection Research Unit (HPRU) in Behavioural Science and Evaluation (BSE) at the University of Bristol.^19^ The project focused on developing dissemination activities with older adults combining three activity types: (i) a questionnaire to ask what kind of activities people aged 55+ might enjoy, (ii) four planning workshops and (iii) four public dissemination events. Our four locations were: Belfast (Northern Ireland, NI), Cardiff (Wales), Glasgow (Scotland) and Tewkesbury (England). We felt it was important to carry out activities across the United Kingdom to support disseminating findings from our UK‐wide research project ‘What does vaccination mean to people aged 55+?’.^20^ The South West England location, Tewkesbury, was selected following feedback from the HPRU BSE Public Involvement Strategy Group, which questioned our plans to hold activities only in major cities. Major cities were otherwise selected in an effort to limit travel requirements.

The questionnaire enabled us to make contact with new potential public contributors, whereas the core PPI activity was planning workshops and following community events. Public contributors were compensated based on £20/h, which was based on local recommendations at the time of applying for project funding in 2020 (see, e.g., People in Health West of England^21^ and National Institute for Health and Care Research^22^). Compensation was offered for reviewing materials and attending the workshops, as well as to those public contributors who helped co‐deliver an activity or who wrote for this publication. We also offered to cover local taxi fares for those who helped co‐deliver the in‐person events.

As this project was set up as public involvement to support the dissemination of research findings, ethical approval was not sought for these activities.^23^ We followed the NIHR's PPI Researcher guidance^24^ on this and it was explained to public contributors that they were advising us how to plan our activities rather than acting as research participants. We followed ethical practices throughout, keeping in mind best practices on confidentiality and opportunities to opt out.^25^ This meant we have only shared details where we have specifically gained permission to use these in publications. We also sent separate invitations for each activity instead of asking contributors to commit to joining all activities at once. The two contributors whose text was included in this paper were asked to write for this specific purpose and written permission was obtained for it to be included in this publication.

### 2.2 Recruiting public contributors

We recruited public contributors on a rolling basis primarily via email and social media by sharing our online questionnaire which also acted as an expression of interest form. We asked organisations and groups to share information with their contacts as well as shared our adverts on Facebook and Twitter. This involved contacting some of the same groups and organisations we had previously been in touch with regarding our UK‐wide research project, which relied on online participant recruitment (for further information, see Silvonen et al.^20^). We contacted local divisions of national organisations such as Age UK as well as a variety of organisations including faith‐based groups across religions, community groups serving a variety of ethnicities and older adults' exercise groups to reach people from different backgrounds. We also shared our online questionnaire (see Section 2.3 and Supporting Information S1: 1) with individuals who had signed up for updates relating to the research project we were aiming to disseminate findings for.

We invited people who expressed their interest in activities on the questionnaire to take part in the online planning workshops in the corresponding areas. Holding the workshops online meant we were able to invite people from two other areas in England to the Tewkesbury workshop and one other area in NI to the Belfast workshop to ensure sufficient turnout and maximise inclusion of diverse perspectives. Only those contributors who attended the online workshops were asked if they would like to help co‐deliver the community events. The invite to attend the events as members of the public was shared with all our contacts.

We hoped to reach people from a range of sociodemographic backgrounds; however, most of our public contributors identified as White British. The contributors were aged 55–84, with the majority falling into the 65–74 age group. Cascading information through large charities such as Age NI, Age Scotland and Age Cymru helped us reach a more diverse group of people. For example, this led to members of an Asian women's group acting as public contributors in our Glasgow planning workshop. Overall, the NI planning workshop was the only one where we were unable to involve public contributors from minority ethnic backgrounds (Table 1).

**Table 1:** Number of public contributors in online planning workshops.

Location	Number of contributors
Tewkesbury/England	8
Cardiff/Wales	7
Belfast/Northern Ireland	8
Glasgow/Scotland	10
Total	33

### 2.3 Online questionnaire

An online questionnaire focusing on what activities older adults might enjoy was shared electronically via MS Forms (see Supporting Information S1: 1). The questionnaire was open to all aged 55+ but stated that some activities would only be available in certain locations. The questionnaire was live from December 2021 to August 2022 and was completed by 121 people. The questionnaire consisted of six questions focusing on the type of individual and group activities people enjoyed as multiple‐choice questions and an open‐ended question. We also asked if people preferred to take part in activities online or in person as remaining coronavirus disease 2019 (COVID‐19) restrictions were lifted in Spring 2022 in the United Kingdom. Only eight people said they would prefer in‐person activities, which likely reflected the circumstances at the time when pandemic restrictions were still in place.

The questionnaire showed that attending talks or debates, reading and visiting exhibitions were some of the preferred general activity options. When asked what group activities people enjoyed, the most popular answer options were a discussion group and a talk with a questions and answers (Q&A) session, followed by ‘coffee, cake and chat’. Those who used the free‐text option to share further suggestions for activities suggested walking‐related activities like rambling. The less common suggestions included drama‐based activities and poetry.

We also asked if the respondents were interested in contributing to further PPI activities, in addition to basic demographic questions. As the purpose of the questionnaire was to generate ideas and to provide a starting point for workshop discussions, questionnaire results were collated and shared with those attending the planning workshops.

### 2.4 Planning workshops

We held the 2‐h planning workshops online using Zoom video calling. Aware of the limitations of this approach due to the requirements for digital access (see e.g., Centre for Ageing Better^26^), we felt it was appropriate to hold the workshops online to limit travel requirements and in‐person contact due to COVID‐19 restrictions. Potential contributors were offered support with Zoom, including a Zoom guidance document.

We shared the updated questionnaire results with the public contributors before each planning workshop. The information was shared via email or sent in the post along with a refreshment pack. The pack included a tea and coffee sachet, biscuits and a dried fruit and nut packet (depending on dietary requirements). Most contributors agreed to receive post from us and were positively surprised by the refreshment pack, which helped create a shared comfort break despite meeting remotely. All planning workshops included an introduction to the project, the research team and PPI; public contributor introductions; discussion of the questionnaire results; discussion about preferred activities followed by specific questions about venues, catering and advertising. Guidance on how to hold planning workshops was sought from local PPI experts. CM also attended half of the first workshop as an observer, providing initial feedback.

The workshops took place in spring (Tewkesbury: 23 February 2022; Cardiff: 14 April 2022) and summer 2022 (Glasgow: 27 July 2022; Belfast: 23 August 2022). These workshops informed how we ran the community events: Cardiff (24 August 2022, following a stall at Grangetown Festival 25 July 2022, see Silvonen and McGrath^27^); Belfast (11 November 2022, coinciding with Belfast City Council's Positive Ageing Month); Glasgow (23 November 2022) and Tewkesbury (26 January 2023).

## 3 RESULTS

### 3.1 Planning workshops

Planning workshops built on questionnaire responses as we shared questionnaire highlights with public contributors in advance and discussed these further during the workshops. The 2‐h planning workshops were divided into sections that were led and facilitated jointly by T. S. and H. C., alternating who was talking and who was noting key points on online Padlets. All workshops included a 15 min break, during which the researchers decided the key points for discussion during the second half of the workshop. The workshops were recorded with verbal consent gained from public contributors at the beginning of each workshop. The recordings were compared with the corresponding Padlets to ensure relevant details were captured before the recordings were deleted. Notes from each workshop were initially revisited when planning the related community event and furthermore compared across all workshops by T. S.

Public contributors were encouraged to share their suggestions and previous experiences of activities in more detail, framed around the questionnaire (see Supporting Information S1: 1). For example, while the questionnaire responses showed that people preferred online activities, the workshop discussions clarified that holding in‐person events in the local community would be preferred if there were no restrictions on group activities at the time. Hybrid options were still supported by many as this would mean there was no need to arrange travel and those who did not live locally would still be able to attend. Concerns about digital access were especially emphasised in our NI and Glasgow workshops. Overall, digital exclusion was considered a reason to offer the option of attending activities in person.

Contributors across all of the workshops felt limiting the length of activities to approximately 2 h in total, accessible venues and good public transport links were important aspects relating to holding in‐person events. While many thought a conventional talk (maximum 20 min) followed by a question and answer session would be the most informative activity, some contributors wanted to see something novel such as combining visual material like posters with a talk. Contributors in all of the four planning workshops suggested that we offer short activities that can be attended in a flexible manner and that we collaborate with other events or groups. All contributors raised concerns regarding the health and safety aspects around COVID‐19 when bringing people together for in‐person activities.

There were not many differences between the input from public contributors across the different locations. In Cardiff, the contributors suggested that holding a ‘have your say session’ would be a good opportunity for people to share their experiences and that it was important to have a quiet breakout space accessible throughout the activities. In both Glasgow and NI, contributors raised concerns about lack of continuity when activities are held on a one–off basis. We also received location‐specific venue suggestions, including that the neutrality of a venue should also be considered in NI due to local history. Overall, aligned with the popularity of the ‘chat and coffee and cake’ option in the questionnaire, the contributors felt that offering nice refreshments or food would be a good enough incentive for people to attend free activities, whereas the contributors in Glasgow suggested offering a goodie bag could encourage people to come along.

### 3.2 Community events

The events were built on suggestions from each planning workshop, which meant that some activities were tailored to the location. However, all four events included a ‘talk with Q&A session’ and our ‘write us a postcard’ activity where we encouraged attendees to write or draw any thoughts or experiences they wanted to share with us relating to our research, health research or PPI more broadly. Following feedback from the workshops, the events were informal events focusing on sharing some findings from our research on vaccination for older adults.^20^

We invited two contributors from each workshop to join us in co‐delivering an activity during the community events; however, this only materialised in Cardiff and Belfast despite our efforts to apply this approach across all locations. The general invite to attend the events was also shared by all who contributed to the planning workshops. Overall, six people who contributed to the workshops attended the events, with a further three contributing to delivering the events in total. All events were advertised through Facebook advertising, local event listing webpages and through local community groups (see Supporting Information S1: 3). The events were free to attend and held at accessible community venues ranging from a community centre to a local theatre. Light food with refreshments was offered at all events. Offering refreshments tailored for the groups we aimed to involve in our events was suggested by local PPI colleagues. The only mention of refreshments in our online questionnaire was the ‘coffee, cake and chat’ option (see section 2.3), which was one of the popular group activity options. We discuss each event separately below, focusing on what worked well and what was particularly challenging.

#### 3.2.1 Cardiff

Holding the Cardiff event in August meant the weather allowed us to hold a short ‘walk and talk’ activity outdoors. Half of the group preferred to stay indoors and chat instead, which shows the importance of offering options for activities and having several facilitators present. The attendees (10 in total) were very engaged with the topic and made comments and questions throughout our talk, leading to a whole hour of talking about our research project in an informal setting.

#### 3.2.2 Belfast

Our event in Belfast included holding an information stall in the morning as part of the Positive Ageing Month event ‘Be Prepared’ arranged by the Belfast City Council. We joined over 20 other stall holders, which brought a broad audience of older adults into a community leisure centre where we held further activities in the afternoon. Holding the stall allowed us to invite people to join our afternoon activities, with in‐person presence providing an opportunity to build some rapport. We also noted that attendees appeared pleased to receive free promotional materials from other stall holders. The main activity of our afternoon was our talk and Q&A, which followed a more conventional presentation setting. The attendees (14 in total) raised questions about the suitability of vaccines when on certain medications, which led to some peer‐to‐peer information sharing. Having sandwiches over hot beverages and socialising was a suitable icebreaker as the light lunch break made the atmosphere feel more familiar.

#### 3.2.3 Glasgow

The turnout at our Glasgow event (three in total) was the lowest of all four events, even though we had a centrally located venue just across one of the main train stations. This shows that adverse circumstances such as bad weather can mean turnout is low, especially when holding a stand‐alone event. We also introduced goodie bags in Glasgow and brought in a light indoor exercise activity. Both were received well by the attendees and the low turnout meant there was ample time for informal chats. This resulted in the attendees sharing tips about which groups to join as well as plans to introduce light exercise from ‘Move it or Lose it’^28^ to other groups.

#### 3.2.4 Tewkesbury

Following positive feedback from our Glasgow event, as well as considering weather conditions in January, we included a light exercise demonstration from local providers Fit for Life.^29^ The attendees (11 in total) were particularly interested in the talk and the opportunity to ask questions—two people attended just this activity. People were especially interested in how the data were analysed. Some asked for percentages of responses, suggesting that qualitative health research was potentially less familiar to them. We also received similar comments at our Cardiff event.

## 4 DISCUSSION

The purpose of this project was to invite older adults to plan and co‐produce dissemination activities together with researchers to create meaningful public engagement events for people aged 55+. The interactions with our public contributors showed that even though many people held active roles in their communities, they had not acted as public contributors in health research nor heard of PPI previously. This along with questions about the purpose of our activities suggests that further awareness‐raising and involvement in health research is needed. Our events also showed that people seemed less familiar with qualitative health research, as some expected more focus on statistics or clinical trials. The feedback also highlighted the importance of being clear about the aims and purpose of the activities from the very start. Ensuring contributors understand what they are asked to contribute is essential also from an ethical perspective as being misinformed hinders transparency in involvement.^25^

To understand how the project was experienced by public contributors who joined both an online planning workshop and helped deliver the in‐person events, we asked two contributors to share some written reflections. To help focus the writing activity, we asked the contributors to write short paragraphs based on what they wanted to share about their experiences, motivations, benefits or barriers to involvement. Our public contributors said:

I was nominated by [a local organisation] to be involved in the project as I know the area well. I have a lot of local knowledge and have been involved in lots of local groups. From my experience in arranging events and getting funding for projects, I can say it's easier to get organisations involved and more difficult to get the people there. It can be difficult to see how things relate to the outside world and how we can utilise our skills when working with people.

The activities I joined and what you try to do is great. It won't work just on its own because projects can't stand alone. The online planning workshop was a very helpful starting point where local people could share their views. It was good that it was then followed up with something in‐person and you were willing to come here and share your work in Cardiff! The involvement in the Grangetown Festival was a positive move. It's important to take it to the streets and make it more authentic with community involvement. The community event was great. Ten people attended it which is a good turnout. It helped that the food was delicious! It was a good place to make connections in the local community.

Maybe you should try and get more connected with local organisations and politicians. It's important to build on local networks but you need to be careful not to be too onerous when people are only volunteers. You should also think about how to maintain a presence. (Public Contributor from Cardiff)

I received the invite to participate in the research via Age NI. My interest in taking part stems from being interested in research generally but I was also curious about why so many people are suspicious about vaccinations. As it was from a university, I felt confident it would be rigorous research and have some purpose and therefore worth contributing to. The financial incentive also drew me as this is unusual and it is nice to be rewarded for my time. It felt like my opinion must be worth something if I was being rewarded—that is very encouraging.

I enjoyed all the bits I was involved in. Taking part in the zoom meeting about how to hold an event presenting the research was quite challenging to me as the scope and purpose seemed a bit abstract but I understood eventually. Seeing the research come to life in person some months later at the actual presentation event in Belfast was enjoyable and interesting. It was in a new venue for me and an area I don't know well so an adventure to go there. I felt that the Positive Ageing event which was attended by many older people was an ideal place to present the research. Listening to other older people talk at our stall about their experiences of vaccinations, the health service etc. was interesting and I enjoyed encouraging them to complete postcards recording their thoughts. Meeting the researchers in person was lovely and having gone through the process from start to finish it felt like I'd been part of the whole project (albeit in a very small way). I'm hoping having someone local there, like me, to encourage people to participate was helpful. (A. Murray, Public contributor from Belfast)

Similar to the reflections provided by the public contributors above, the researchers felt the approach improved throughout the project as we learnt more from the people involved in the activities. As our three activities were linked, we felt that we were able to gradually understand what works and what we should do differently. On the other hand, contributing to activities over a period of time also seemed beneficial to public contributors, bringing clarity to topics that at first can seem abstract, which was highlighted in the reflection written by the public contributor from Belfast. These reflections are aligned with previous work that suggests that sharing responsibilities between older adults and researchers facilitates building trust.^30^ While older adults' have been shown to wish to have an active role in health care,^31^ ‘negative attitudes and beliefs about their role’ as a patient can overshadow actual abilities and potentially hinder participation in health activities.^4^

We also recognise the value of collaborating with existing groups, as mentioned in the reflection by the public contributor from Cardiff. Working with existing groups and building connections with public contributors can support efforts to reach diverse audiences. We aimed to involve older adults from ethnically and sociodemographically diverse backgrounds throughout this project, yet our approach to contact community groups working with different faith and ethnic groups was only able to deliver limited results. While most of our activities attracted some people from different socioeconomic and ethnic backgrounds, our planning workshop contributors in NI were all white British. To further improve diversity, deeper connections with existing communities would have been needed. Potential ways to establish this can be partnering with trusted community leaders^32^ and tailoring events more to different cultural needs.^33^ However, limited resources and working across four locations where we had few connections meant we were only able to work with existing groups to a limited extent.

Our multiphase approach—the questionnaire informing the workshops that shaped our events—helped us consider the balance between information and entertainment in public engagement events. Before starting this project, the researchers involved had limited experience of undertaking PPI. Our preconception was that engagement events should include an element of creativity. This was based on previous literature, which shows creative examples of engagement activities: the use of art galleries in health intervention dissemination,^34^ as well as drawing activities as a way to engage adult audiences^35^ or improve accessibility of research outputs.^4^ We also encountered few publications sharing PPI work that focused on older adults beyond specific conditions such as Alzheimer's,^7^ other forms of dementia^36^ or issues such as frailty,^30^ all of which focused on information sharing. Even though some of the contributors enjoyed arts and crafts or drama and poetry, most of the people we spoke to found these types of activities off‐putting in a group setting. Instead, we found that light physical activities, such as doing a ‘walk and talk’ activity or an exercise demonstration, brought something different to our events. While we were only able to do the ‘walk and talk’ at our Cardiff event due to weather, the light exercise aspect was enjoyed by the attendees across all our events, which is why we encourage others to consider similar activities when engaging with older adults.

One of the key messages regarding planning events was to tailor the activities to each location and include some socialising. The contributors also emphasised that offering different ways to take part is important so that people could choose activities based on individual interests, taking into account that older adults are not a homogeneous group of people. While there may be some needs‐based similarities such as access to transport—as suggested by the discussions in our planning workshops—people aged 55+ includes a broad range of people. Two very different reflections from our contributors in Cardiff and Belfast emphasise this, showing the wealth of experiences people aged 55+ can bring to PPI. It should also be noted that older adults may not consider themselves to be ‘old’.^12^

The novelty of this project lies in its focus on dissemination activities, especially how public involvement activities with older adults helped shape engagement with people aged 55+ years. Our multilocation approach facilitated dissemination across the United Kingdom, which was important to us to ensure we could share the findings of previously completed UK‐wide research as widely as possible. Overall, our PPI activities raised awareness of both health research and PPI opportunities among older adults. Our multiphase approach helped us understand what approach to take with dissemination events, for example, moving away from online activities based on the discussions in our planning workshops. Whereas the reflections from our public contributors highlight that some of our online discussions were somewhat abstract, both gave positive feedback for in‐person activities. Previous literature also suggests that in‐person dissemination activities are more suitable for ensuring members of the public understand research findings due to the opportunities for dialogue with researchers.^16^ While it is important for PPI to focus also on research design and preparation,^12^, ^14^ a holistic approach that also considers the relevance of PPI in planning dissemination activities to support wider engagement is recommendable. Thoroughly planned dissemination also helps communicate how contributors and participants have shaped a project.

## 5 LIMITATIONS

Low turnout at some of the events limited the potential to share our work, even though smaller audiences made it possible to have more personal discussions. We also did not explicitly ask permission to publish information from the questionnaire, which has caused some challenges in sharing the findings of this project, in addition to highlighting the importance of following ethical practices in PPI. The public contributors who joined the planning workshops often had very active roles in their communities. This meant that we engaged with few people who are usually not involved in local matters even if the PPI experience as a whole was new to all our contributors. We also did not explore the possibility of having a medical professional join our events, whereas attendees often had specific medical questions that the researchers were unable to answer.

The PPI activities were spread over 14 months in total. This limited continuous engagement, however, most public contributors still continued their involvement despite a long break. Overall, the extended duration of the project meant we were able to apply learning as we gradually proceeded with the project.

## 6 CONCLUSION

The ageing population in the United Kingdom means an increasing number of older adults are using healthcare services.^3^ This increased use should be reflected also in the involvement of older adults in health research as public contributors, which is why opportunities to collaborate with and seek feedback from people aged 55+ are advised. We hoped to promote inclusivity and advance the understanding of older adults as a broad age group and suggest that projects consider working more closely with existing communities to support the inclusion of underrepresented groups. Based on the interactions we had with members of the public, we suggest that PPI activities involving older adults should be informative whilst providing opportunities to socialise.

Overall, the feedback we gained from our in‐person events was positive and the attendees were enthusiastic about our work even if we did not attract large audiences. We hope that those who did join the PPI activities have gone on to talk about them, further raising awareness of the different ways older adults can be more involved in health research.

## AUTHOR CONTRIBUTIONS

**Taru Silvonen:** Conceptualisation (lead); funding acquisition (lead); investigation (equal); methodology (lead); project administration (lead); resources (lead); writing—original draft (lead); writing—review and editing (equal). **Carmel McGrath:** Methodology (supporting); supervision (lead); writing—review and editing (equal). **Anne Murray:** Investigation (supporting); writing—original draft (supporting); writing—review and editing (equal). **Hannah Christensen:** Conceptualisation (supporting); funding acquisition (supporting); investigation (equal); methodology (supporting); project administration (supporting); writing—review and editing (equal).

## CONFLICT OF INTEREST STATEMENT

The authors declare no conflict of interest.

## ETHICS STATEMENT

Ethical approval has not been sought for this project due to this being a patient and public involvement project.

## Supporting information

Supporting information.

## Data Availability

Data sharing is not applicable to this article as no new research data were created, collected or analysed in this study.
