# Supplementary material for: Reflections from the ‘Hold the door open’ project: Inviting older adults across the UK to shape dissemination of health research findings
Source: Health Expect. 2023 Nov 29;27(1):e13928. doi: 10.1111/hex.13928 (PMC10726280; doi:10.1111/hex.13928)

Supplement 1 Online questionnaire

Community involvement in health research

**Who are we?**
Hannah Christensen and Taru Silvonen are researchers at the University of Bristol.

**What is our project about?**
We are looking for new ways to involve people aged 55+ in health research. This relates to our research project ‘What does vaccination mean to people aged 55+?’ which looks at people's views on vaccination. We are now hoping to share our research results with the general public through community involvement activities.

**What is community involvement?**
We believe community involvement includes open discussions with you about our research so that we can listen, understand and act on your views and opinions. In this questionnaire we ask what kind of activities you and others might enjoy to support those discussions.

**What is this questionnaire about?**
This questionnaire should take no more than 5 minutes to complete.
In this questionnaire, we ask what kind of community involvement activities you might like to be involved in to support open discussions about health research.

Our aim is to be inclusive of different people as well as different opinions. To help us do this, we ask you to share some details about yourself like your age, ethnic group and the first part of your postcode.

This questionnaire is also a way for you to tell us if you would like to:
·       Attend a planning workshop
·       Help us set up a community event or
·       Write with us about these activities

**Payment**
Payment (£20 high street voucher per hour) is offered for taking part in the planning, delivery and writing up of the community involvement activities.
If you’d like to know more you can let us know in the questionnaire. You can also find more information on our project blog: https://healthinvolvement.blogs.bristol.ac.uk

**Data protection**
The answers you provide will be confidential and any personal information is held securely following General Data Protection Regulations.

Thanks for taking the time to share your views with us!

*Required

1. How would you prefer to join activities?*

- Online (using video calling)
- In person
- Some activities online and some in person
- Other:

2. What type of activities do you like?*

- Attending talks or debates
- Crafts using different materials
- Painting or drawing
- Reading
- Making videos
- Visiting exhibitions
- Taking photos
- Writing
- None of these
- Other:

3. What group activities do you like?*

- Discussion group
- A talk with a questions and answers session and refreshments
- Drama-based games and activities
- Using smartphones to share and discuss materials
- Light physical activities
- Coffee, cake and chat
- None of these
- Other:

4. Are there any other activities that you would like us to consider?

5. We plan to use this questionnaire in locations across the UK. If you have any thoughts on how we could improve the questions for when we use it next, please tell us here.

6. Are you interested in hearing more about opportunities to join our researchers in community involvement activities if these are available online or in your area? Please tick all that may interest you

- Planning workshop (online or in-person)
- Co-delivering a community event (online or in-person)
- Writing about the community engagement project (online)

Personal details

It will help us to include people from all different backgrounds and areas if we know a little about you. Please fill in your contact details if you would like us to get in touch with you.

7. What is your age?*

8. Which of the following best describes you?*

- Female
- Male
- Non-binary
- Transgender
- Intersex
- Prefer not to say
- Other:

9. Which ethnic group best describes you?*

- Asian
- Black
- Mixed ethnicity
- White
- Any other ethnic group
- Prefer not to say
- Other:

10. What is the first part of your postcode?*

11. Do you agree to being contacted by a researcher from the (withheld for peer review) to discuss community involvement activities in health research?*

- Yes
- No

12. What is your name and preferred title?*

13. What is your email address?

14. If you prefer to be contacted by phone, please provide your phone number and times when you would like us to call you.

15. Please let us know if you have any requirements or requests to help us support you take part in online or face-to-face activities.

Thank you for completing this questionnaire!

Thank you for sharing your thoughts on community involvement activities!

If you have any questions please contact the research team via grp-vaccinesinolderadults@groups.bristol.ac.uk or by phone 0117 455 7657.

To find out more about the community involvement project please visit our project blog: https://healthinvolvement.blogs.bristol.ac.uk

For more information on our research project, please visit:

http://www.hprubse.nihr.ac.uk/research/research-topics/immunisation-and-vaccination/what-does-vaccination-mean-to-people-aged-55

The information on this form is collected for the ‘Hold the door open – involving older adults from diverse backgrounds in health research’ community involvement project funded by the Health Protection Research Unit in Behavioural Science and Evaluation Community Involvement Scheme at University of Bristol.

Supplement 2 – Example of advert used for contributor recruitment

Figure 1 Example of advert used for public contributor recruitment. Published over Twitter (content by TS).


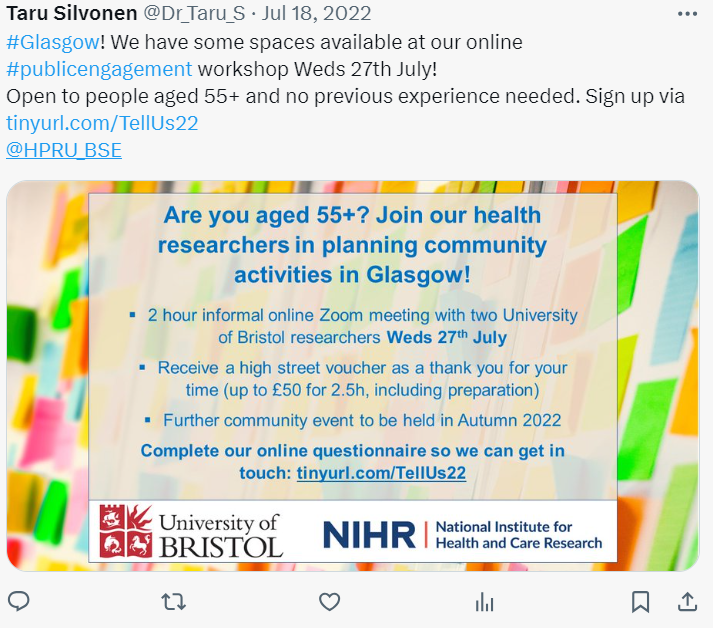


Supplement 3 – Example of advert used for dissemination events

Figure 2 Example of advert used to advertise community events. Published over Twitter (content by TS).


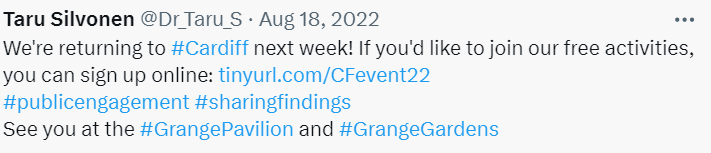


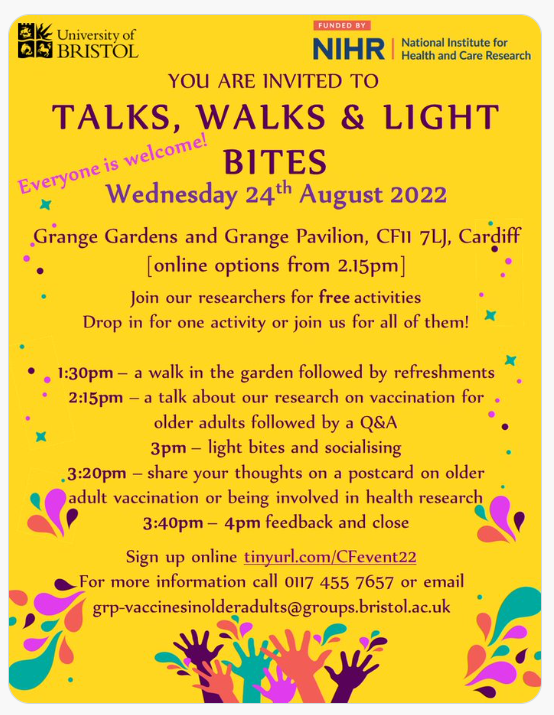

Supplement: Supplementary file 1 — Supporting information. [file HEX-27-e13928-s001.docx]
